# Psychological outcomes following digital replantation and revascularization: A narrative review

**DOI:** 10.1016/j.jham.2026.100492

**Published:** 2026-08-10

**Authors:** Diana Mastellone, Dev Patel, Kenyon Graham, Michael J. Franco

**Affiliations:** a Cooper Medical School of Rowan University, 401 Broadway, Camden, NJ, 08103, USA; b Department of Hand & Reconstructive Surgery Cooper University Hospital, 1 Cooper Plaza, Camden, NJ, 08103, USA

## Abstract

**Purpose:**

Literature on digital replantation has historically prioritized primary outcomes such as viability rates, return-to-work timelines, and restoration of range of motion and grip strength. Psychological and psychosocial sequelae, including post-traumatic stress disorder (PTSD), depression, anxiety, sleep disturbance, adjustment disorder, and body image disruption, are reported inconsistently, infrequently, or not at all. This narrative review examines the scope and quality of psychological outcome reporting in the digital replantation and revascularization literature from 2015 to 2025.

**Methods:**

A literature search of PubMed/MEDLINE, EMBASE, PsycINFO, CINAHL, Web of Science, and the Cochrane Library was conducted for English-language studies published between January 2015 and December 2025, using combinations of terms including “replantation,” “revascularization”, “digit amputation”, “PTSD”, “anxiety”, “depression”, “adjustment disorder”, “body image”, and “psychosocial outcomes”. Studies reporting at least one psychological or psychiatric outcome following digital or upper extremity replantation, revascularization, or upper extremity amputation were included. Adult or mixed adult-pediatric cohorts were included. Given study heterogeneity, findings were synthesized narratively, to broadly characterize the available literature, by outcome domain.

**Results:**

Nineteen studies met inclusion criteria. Depression was the most frequently reported discrete outcome (10/19 studies, 52%), followed by anxiety (8/19 studies, 42%) and PTSD (7/19 studies, 36%). PTSD prevalence ranged from 46% to 69%, and depression and anxiety prevalence ranged from 7% to 71% across studies using validated instruments. Adjustment disorder was identified in two studies, including one large retrospective analysis (n = 1986) demonstrating significantly higher prevalence following replantation failure (2.7% vs <1%). Pre-injury anxiety was identified as an independent predictor of replantation failure in one prospective cohort. No included study reported formal psychiatric or behavioral health referral rates.

**Conclusions:**

There is a pervasive gap in the reporting of psychological and psychosocial outcomes following digital replantation and revascularization. Existing evidence demonstrates that psychiatric morbidity is more common than is currently reflected in traditional surgical outcome reporting. Standardized, validated psychological screening instruments should be incorporated into replantation outcome reporting, and multidisciplinary care models inclusive of behavioral health are warranted.

## 1 Introduction

Traumatic digit amputation affects an estimated 45,000 individuals annually in the United States, with the majority occurring in young, working-age men employed in manual labor.^1^ Since Komatsu and Tamai's first successful digital replantation in 1968, microsurgical technique has advanced to yield viability rates of 80% to 90% at high-volume centers.^2^^,^^3^ Yet the psychological impact of digital replantation, including the traumatic nature of the injury, prolonged recovery, altered body image, and occupational disruption, remain comparatively underreported in hand surgery literature.

The hand is central to identity, occupation, intimacy, and self-expression. Its traumatic loss or disfigurement carries profound psychosocial consequences that functional metrics such as grip strength and two-point discrimination cannot capture. Traumatic injury is a recognized precipitant of post-traumatic stress disorder (PTSD), major depressive disorder (MDD), generalized anxiety disorder (GAD), and adjustment disorder.^4^ Upper extremity injuries are particularly challenging, given their visibility, functional and vocational impact, and the commonly sudden, unexpected nature of the traumatic event.^5^

The paucity of consideration for psychological impacts of replantation is not without clinical consequences. Pre-injury anxiety has been identified as an independent predictor of replantation failure, which is just one potential mechanism by which psychological outcomes affect physical ones.^6^ The impact appears to be bidirectional as well; failed replantation is associated with significantly higher rates of adjustment disorder.^7^ This interplay demonstrates the importance of evaluating psychological outcomes in replantation studies, as patient wellbeing may have an impact on post-surgical success.

This narrative review aims to characterize the current state of psychological and psychosocial outcome reporting in the digital replantation and revascularization literature from 2015 to 2025, incorporating relevant studies of traumatic upper extremity amputation where replantation-specific data were limited, to highlight the need for standardized and validated psychological outcome measures in future replantation research, and to state the need for multidisciplinary follow-up in the perioperative period following upper extremity replantation.

## 2 Methods

A literature search of PubMed/MEDLINE, EMBASE, PsycINFO, CINAHL, Web of Science, and the Cochrane Library was conducted using combinations of the following terms: (Replantation OR revascularization OR digit replantation OR finger replantation OR thumb replantation OR hand replantation OR digit amputation) AND (PTSD OR post-traumatic stress OR posttraumatic stress OR anxiety OR depression OR adjustment disorder OR psychosocial OR psychological OR psychiatric OR mental health OR body image OR sleep OR quality of life) AND (outcomes OR patient-reported outcomes OR sequelae OR burden). Eligible articles were limited to those published in English between January 1, 2015, and December 31, 2025.

Studies were included if (1) they reported outcomes of digital or upper extremity replantation or revascularization or traumatic upper extremity amputation; (2) there was at least one psychological or psychiatric outcome variable reported; (3) participants included adults (18 years or older) or mixed adult-pediatric cohorts; and (4) were published between January 2015 and December 2025. Editorials, case reports with fewer than five patients, letters without original data, and studies focused exclusively on lower extremity or military populations without an upper extremity replantation subgroup were excluded.

Given the heterogeneity of study designs, populations, and outcome measures identified across the literature, a quantitative meta-analysis was not undertaken. A formal study quality assessment was not undertaken in this narrative review, but the authors did consider differences in study design, sample size, and methodological limitations when interpreting the body of evidence. Findings are synthesized narratively, rather than as a systematic review, and organized by psychological outcome domain: PTSD, depression, anxiety, adjustment disorder, sleep disturbance, body image and psychosocial adaptation, and the role of pre-existing psychiatric morbidity.

## 3 Results

The literature review yielded 19 articles reporting psychological or psychosocial outcomes in the context of digital replantation or revascularization, published between 2015 and 2025. Studies originated from the United States, China, Turkey, Nordic countries, Australia, Germany, Canada, and Indonesia, reflecting a geographically broad and methodologically heterogeneous body of evidence. Study designs included retrospective cohorts, prospective cohorts, cross-sectional surveys, systematic and integrative reviews, with varying sample sizes (Table 1). Across the literature, a consistent pattern emerges, psychological outcomes are infrequently prioritized and inconsistently standardized with instruments. Despite these limitations, the available evidence collectively demonstrates a substantial and largely unaddressed psychiatric burden in this population. The following sections synthesize findings by respective psychological outcome domains.

**Table 1:** Characteristics of included studies reporting psychological or psychosocial outcomes following digital replantation or revascularization (2015–2025).

Reference Number	Author, Year	Journal	n	Design	Psychological Outcome	Psych Instrument(s)
6	Jin H et al., 2019	International Journal of Surgery	102	Prospective cohort	Anxiety, Depression	HADS, SAS, SDS
7	Miller BN et al., 2025	Plastic and Reconstructive Surgery - Global Open	1986	Retrospective	Adjustment disorder	ICD-10 coding
8	Moltaji S et al., 2020	Journal of Hand and Microsurgery	56 articles	Systematic review	Quality of life	BDI, Screen for Posttraumatic Stress Symptoms (SPTSS)
9	Yoon AP et al., 2021	Journal of Hand Surgery	280	Retrospective	Patient wellbeing	MHQ, SF-36
10	Shue S et al., 2021	Journal of Hand and Microsurgery	46	Retrospective cohort	MDD, PTSD, Adjustment disorder, Anxiety, Panic disorder	ICD-10 coding, DSM-5 criteria
11	Cohen-Tanugi S et al., 2024	Hand	39	Retrospective Observational Single-center Cohort	PTSD, Depression	PC-PTSD, CES-D, VR-12
12	Maddison K et al., 2022	Journal of Hand Surgery [European Volume]	503 (9 articles)	Integrative review	Depression, Anxiety, PTSD	HADS, IES, CES-D
13	Minnucci S et al., 2025	Journal of Hand Therapy	3315 (21 articles)	Systematic review	Depression, Anxiety, Catastrophizing, Kinesiophobia	Multiple validated
14	Ladds E et al., 2017	Journal of Hand Therapy	1642 (19 articles)	Systematic review	PTSD, Depression, Anxiety	IES-R, BDI, HADS, IES, ASI, DAAS, PASS, STAI, SRS-PTSD, SPSS, PSS, CESDS, PNAS
15	Güvenç Ket al. 2025	Journal of Plastic Surgery and Hand Surgery	95	Cross-sectional	Depression, Anxiety, Sleep Quality	BAI, BDI, PSQI
16	Ge L et al., 2026	Frontiers in Neuroscience	96	Prospective cohort	PTSD, Inflammatory markers	PCL-C
17	Ghițan AF et al., 2023	Brain Sciences	166	Case-control	PTSD	PCL-5
18	Ghițan AF et al., 2023	Current Health Sciences Journal	13 articles	Retrospective	Anxiety, Depression, PTSD	Qualitative psychological outcomes
19	Rosberg HE et al., 2022	Hand Therapy	9	Qualitative	Body image, Psychosocial adaptation	Semi-structured interviews (content analysis)
20	McDonald S et al., 2021	Frontiers in Psychology	227	Cross-sectional	Anxiety, Depression, Quality of life, Body image	HADS, WHOQOL, ASI-R, BIDQ
21	DiGiovanni PLC et al., 2024	Hand Surgery and Rehabilitation	36	Cross-sectional	Depression	PROMIS Depression
22	Pyörny J et al., 2024	Clinical Orthopedics and Related Research	254	Retrospective	Quality of life	EQ-5D-5L, CISS, MHQ
23	Naylor JM et al., 2023	BMC Musculoskeletal Disorders	386	Prospective cohort	Patient-perceived global mental health, Quality of Life	Global mental health, EQ-5D-5L
24	Kulkarni A et al., 2024	Journal of Hand Surgery [American Volume]	NA	Descriptive	NA	NA

### 3.1 Primary finding: psychological outcomes in 19 studies

The principal finding of this narrative review is that psychological and psychosocial outcomes were reported in 19 studies.

From the available data, psychological consequences of replantation are prevalent, varied, and underreported. The Finger Replantation and Amputation Challenges in Assessing Impairment, Satisfaction, and Effectiveness (FRANCHISE) multicenter international cohort identified that revision amputations may take up to 4 years to achieve recovery from psychological trauma, though recovery was not as prolonged in the replantation group, based on MHQ and 36-Item Short Form Survey (SF-36) trends, which is often longer than functional recovery times.^8^ An analysis of isolated traumatic upper extremity amputations reported a new onset of psychiatric diagnosis (MDD, PTSD, Adjustment Disorder, GAD, or Panic Disorder) in 67.4% of the study population.^9^

### 3.2 Depression

Depression was the most frequently reported discrete psychological outcome, reported in 10 included studies (52%). Cohen-Tanugi and colleagues found that 51% of traumatic upper extremity amputees screened positive for depression, at a median of 17 months, using the Center for Epidemiologic Studies Depression Scale (CES-D).^10^ The physical component score of the Veterans RAND 12-Item Health Survey (VR-12), representing self-reported physical health, was significantly worse for patients with depression.^10^

An additional review analyzed the psychological impact of traumatic hand injuries from 2008 to 2020, reporting depression prevalence of 7% to 71% in included studies.^11^ Minnucci and colleagues' 2025 systematic review of 21 longitudinal studies (n = 3315) found that depression was independently associated with worse pain, greater disability, reduced grip strength, impaired range of motion, and lower return-to-work rates, reinforcing the physical consequences of poor mental health following replantation.^12^

### 3.3 Anxiety

Anxiety was tied for second most frequently reported discrete psychological outcome, with 8 out of 19 (42%) studies reporting it in their findings. Post-injury anxiety prevalence in hand trauma populations ranges from 23% to 71% across studies, significantly higher than in the general population.^13^ Ladds and colleagues' systematic review identified that post-injury anxiety declined over the 18 months follow up, and that persistence of symptoms after 3 months was a strong predictor for chronicity.^13^

Multiple studies looked at the physical effect of anxiety on replantation success. Jin and colleagues’ prospective cohort study of 102 patients (134 digits) found pre-injury anxiety, measured by the Hospital Anxiety and Depression Scale (HADS-A), to be an independent risk factor for digit replantation failure.^6^ Notably, double arterial anastomoses increased replant survival specifically in patients with elevated pre-injury anxiety, suggesting a potential operative mitigation strategy while further emphasizing the importance of evaluating and addressing the psychological impact of traumatic injuries.^6^ This represents the only published prospective investigation of a psychological variable as a direct predictor of surgical outcome in replantation.

Guvenc and Guvenc's 2025 study directly compared anxiety, depression, and sleep quality between patients with successful replantation and those with finger loss following failed replantation or local flap closure. They utilized the Beck Anxiety Inventory, Beck Depression Inventory, Pittsburgh Sleep Quality Index (PSQI), and Quick Disabilities of the Arm, Shoulder and Hand questionnaire (QuickDASH). The successful replantation group showed significantly better outcomes across all measures, reinforcing the negative relationship between traumatic replantation failure and worse psychological outcomes.^14^

### 3.4 PTSD

PTSD was the third most consistently reported discrete psychological outcome, it was reported in 7 included studies (36%). Cohen-Tanugi and colleagues found that among 39 adults with surgically managed traumatic upper extremity amputation, 69% screened positive for PTSD at a median follow-up of 17 months using the Primary Care PTSD Screen (PC-PTSD), with a median time to first positive screen of 10 months.^10^ This delay may suggest that shorter follow-up periods characteristic of most replantation studies lead to systemic PTSD underdiagnosis.

A prospective cohort of 96 digit replantation patients were assessed for PTSD using the validated PTSD Checklist-Civilian Version (PCL-C) at 7 days postoperatively, with 44 patients (45.83%) meeting criteria. Female gender, lower education level, complete amputation, dominant hand involvement, and greater number of amputated digits were significant predictors for PTSD (p < 0.05). This study also correlated PTSD severity with serum inflammatory markers (IFN-γ, TNF-α, IL-1β, IL-6, IL-10), identifying potential biological correlates for early identification of at-risk patients.^15^

Two studies by Ghițan and colleagues examined PTSD in hand trauma patients. In their case-control study, 166 individuals with complex hand and forearm injuries were assessed three months post-surgery using the PCL-5. They found significant associations between PTSD symptoms, female sex, lower socioeconomic group, and lower educational attainment.^16^ An additional review by this same group assessed risk factors and psychological outcomes in complex hand trauma patients and the role of a multidisciplinary approach. They identified anxiety, depression, and PTSD as the primary psychological effects, stemming from both the traumatic event itself and secondary challenges like loss of independence and reduced ability to perform daily activities.^17^ Taken together, these two papers include PTSD with several overlapping psychological sequelae of hand trauma that warrant routine screening, and coordinated multidisciplinary care.

### 3.5 Adjustment disorder

Adjustment disorder was explicitly identified in two retrospective studies. In Miller et al. of the 516 patients experiencing replantation failure, the prevalence of an adjustment disorder diagnosis was 2.7% versus less than 1% in the 1470 patients with successful replantation (p = 0.02).^7^ In Shue et al. adjustment disorder was present in 11 of 31 patients, and was the third most prevalent psychological diagnosis after MDD and PTSD, following traumatic upper limb loss.^9^

Although diagnosis coding and the retrospective design of this study are limitations, these findings suggest that failed replantation may be more psychologically traumatic than the original mechanism of injury, though more studies investigating adjustment disorder are needed. The multitude of negative psychological sequelae in digital replantation underscores the importance of multidisciplinary care in this subset of the replantation patient population.

### 3.6 Sleep disturbance

Sleep disturbance was measured as a discrete outcome in only one study, using the validated PSQI. In Guvenc's 2025 study, sleep quality was significantly higher in the successful replantation group than the failed replantation group (p < 0.001).^14^ This represents the only replantation-specific cohort study within the inclusion period to assess sleep quality. Sleep disturbance is well-documented as a component of PTSD and depression phenomenology and as an independent cause of morbidity following traumatic injury, yet its longitudinal course and impact on recovery remain largely unstudied.

### 3.7 Body image, identity, and psychosocial adaptation

A Swedish qualitative study of nine patients undergoing replantation during young adulthood is the only study within the inclusion period to examine body image and psychosocial identity. Semi-structured interviews identified five emergent themes including memories of the injury and concerns for the future; hand function, pain, and cold sensitivity; feelings about having a visibly different hand; adaptation to impairments and challenges in daily life; and messages to healthcare professionals. This article highlights the importance of patient-centered care and including ongoing emotional support to reduce negative implications in body image and identity.^18^ A key limitation to generalizing the results of this study was the adolescent study population, as adolescence is already a time period where concerns over body image surface, it is unknown if this trend would be as prevalent in adult populations.

McDonald and colleagues' review of body image in amputees found that individuals with visible bodily changes experienced the poorest body image scores, which were associated with poorer psychosocial outcomes including depression, anxiety, and reduced quality of life.^19^ DiGiovanni and colleagues applied Patient-Reported Outcomes Measurement Information System (PROMIS) instruments focused on mental health, physical health, and depression to a replantation cohort (n = 36, median follow-up 6.1 years). They found that while upper extremity function remained below population norms (median PROMIS Upper Extremity Function score 40.6 vs. 50.0), participants reported global mental health and depression scores comparable to the general population.^20^ These findings suggest that self-perceived mental health post replantation may resemble that of the greater population, with increased time after successful replantation. However, patients with neuroma experienced a statistically significant decrease in mental well-being score (p < 0.05), highlighting that ongoing replantation complications may disrupt the process of psychological improvement over time.^20^

In a retrospective study of traumatic upper extremity amputations, replantation over revision amputation was not associated with improvements in patient-reported hand function, quality of life, or hand aesthetics.^21^ This study challenges the assumption that many surgeons operate on, that replantation is superior to revision amputation, and thus reinforces the need for more research that investigates the psychological impact of replantation, to better guide patient care.

### 3.8 Pre-existing psychiatric morbidity as a moderator

Naylor and colleagues conducted the only study that examined the impact of pre-morbid mental health diagnosis on outcomes following traumatic hand injury surgery (tendon injuries, fractures, amputations etc.). In a multicultural cohort assessed at baseline and 3 months postoperatively, patients with a pre-morbid mental health diagnosis had lower odds of reporting ‘good’ mental health at follow-up (OR 0.23, 95% Confidence Interval (0.18, 0.47), p < 0.001), independent of injury severity.^22^ This reinforces that psychiatric comorbidity is a modifier of recovery trajectory and should inform treatment.

### 3.9 Formal psychiatric and behavioral health referral

One of the most striking findings of this review is the absence of any data on formal psychiatric or behavioral health referral rates following replantation. None of the included studies reported what proportion of patients were referred to psychiatry, psychology, or social work after replantation or replantation failure.

The exception to the lack of formal psychiatric referral was the multidisciplinary care framework described by Kulkarni and colleagues, who presented a blueprint for an upper extremity limb loss clinic incorporating preoperative psychology and psychiatry evaluation as a standard protocol.^23^ This represents a novel paradigmatic shift in management following upper extremity loss, though the results of this study are limited in that they did not track whether this intervention improved psychological outcomes. This study also has limited generalizability to all patients undergoing reconstruction as it only included patients with scheduled surgery, not traumatic injury and acute replantation.

## 4 Discussion

This narrative review reveals a critical and pervasive gap in the reporting of psychological and psychosocial outcomes following amputation, digital replantation and revascularization. Across more than a decade of published studies, psychological outcomes were only present in a small number of relevant studies. No study reported psychiatric referral rates. Psychiatric instruments were heterogeneous, rarely validated, and inconsistently timed. The result is literature that can describe with considerable precision the arc of tendon healing and grip strength recovery, but cannot tell us what proportion of replantation patients develop PTSD, how many receive psychological care, or whether successful replantation mitigates psychiatric morbidity relative to revision amputation. This gap is not insignificant. PTSD rates approaching 46% to 69%, depression rates of 51%, and the identification of pre-injury anxiety as an independent predictor of replantation failure suggest a multidimensional psychological concern in digit amputation and replantation.^6^^,^^10^ It may be central to the very outcomes surgeons care most about, namely, survival of the replant, functional recovery, and return to work.

Adjustment disorder is defined as the development of emotional or behavioral symptoms in response to an identifiable stressor.^24^ Despite adjustment disorder being the most clinically immediate psychiatric diagnosis following a trauma, it is largely absent in outcomes analyzed in surgical literature.

### 4.1 The physical-psychological interaction

Several of the included studies identified bidirectional relationships between psychological and functional outcomes. Jin and colleagues' finding that pre-injury anxiety predicts replantation failure represents a possible mechanism of anxiety-mediated sympathetic activation that may contribute to physiological changes that could adversely affect microvascular outcomes, though this mechanism remains theoretical. This, taken with the findings of Ge's article, that there is a correlation between PTSD severity and serum inflammatory markers, provides a theoretical association between psychological distress and post-operative outcomes, although additional studies are required.^6^^,^^15^ If confirmed in larger prospective studies, this would argue for psychological screening not only as a postoperative measure but as a preoperative surgical risk stratification tool.

Additionally, data from Minnucci and colleagues' systematic review of 3315 hand and wrist injury patients confirms that post-injury depression, anxiety, catastrophizing, and kinesiophobia independently predict worse functional outcomes across multiple domains.^12^ This study suggests that unaddressed psychological morbidity may perpetuate a cycle of impaired functional recovery and worsening psychological status, ultimately contributing to functional impairment.

### 4.2 The measurement problem

Out of the 87 distinct measurement instruments identified by Moltaji and colleagues' systematic review, only eight were validated and reliable, which reflects a lack of standardized core outcomes in replantation research.^25^ The psychological domain is largely the target of this shortfall. The instruments that appear across studies (BDI, HADS, CES-D, PCL-C, PROMIS Depression) are all validated, but are used inconsistently, at variable time points, and in disparate populations, precluding pooled analysis. The field of trauma psychology has established brief, validated instruments for each relevant construct: the PCL-5 for PTSD; the PHQ-9 for depression; the GAD-7 for anxiety; the PSQI for sleep; and PROMIS for multidimensional patient-reported health.^26^, ^27^, ^28^ Their incorporation en masse into replantation outcome studies requires minimal additional patient burden, and significant benefits for physicians, researchers, and ultimately patients.

### 4.3 Replantation failure and compounded traumatic burden

Replant failure is an especially distressing complication, layering an additional stressor onto an already difficult experience: major surgery, fear of losing the replanted part, and then the eventual amputation. This cumulative burden raises psychological risk and is associated with higher rates of adjustment disorder diagnoses.^7^

The Guvenc study's findings of significantly worse anxiety, depression, and sleep quality in patients with digit loss versus successful replantation provides preliminary evidence that replantation success may be psychologically protective, but prospective confirmation with pre- and postoperative measurement designs is a critical goal of future research.^14^

### 4.4 Evidence based recommendations for clinical practice

Based on the authors’ interpretation of the available evidence, the following practices may help guide future multidisciplinary care, but are not evidence-based guidelines:

● Consider preoperative assessment of anxiety, or history of mental health diagnoses, using validated scales, when feasible
● Consider screening for PTSD at approximately 6 and 12 months following replantation
● Consider periodic screening for depression and anxiety in the postoperative period, and for patients with a positive screen consider offering additional psychological support
● Provide ongoing emotional support and acknowledge the psychological impact of traumatic upper-extremity injuries
● Institutions may consider developing structured pathways for behavioral health referral, and when possible use a multidisciplinary approach involving mental health professionals

### 4.5 Evidence based recommendations for future research

Future replantation outcome studies should consider incorporating a standardized minimum psychological outcome dataset. This framework is proposed by the authors as informed by the reviewed literature, it is not a validated or evidence-based guideline. We propose the following reporting standard: PCL-5 at ≥6 months for PTSD; PHQ-9 at 3, 6, and 12 months for depression; GAD-7 at 3, 6, and 12 months for anxiety; PSQI at 6 and 12 months for sleep; and PROMIS Global Health and Upper Extremity at 6 and 12 months. Behavioral health referral should be documented as a binary outcome for all patients. Pre-operative baseline psychological assessment should be incorporated into prospective studies to establish the natural history of psychological morbidity following replantation and to test whether replantation success independently confers psychological benefit relative to revision amputation. Incorporating these psychological outcomes allows may allow for a more comprehensive assessment and care of replantation patients.

### 4.6 Limitations

This review has several limitations. This was an intentionally broad narrative review; as such formal systematic review methodology including PRISMA reporting and risk of bias assessment was not performed. The available literature varied considerably in study design, cohort size, psychological outcome measures, and overall methodological quality. The narrative approach allowed for inclusion of the limited literature on this topic, though this heterogeneity precluded meta-analysis, limited generalizability, and increased the potential for selection and measurement bias. As such, the reported prevalence of outcomes should be interpreted with caution, as the findings of this review may be more useful in identifying knowledge gaps and consistent themes, rather than precisely estimating the prevalence of psychological outcomes following replantation. This review is also limited by inclusion of studies that were not exclusively replantation cohorts. Additionally, the 2015 to 2025 search period excludes relevant foundational works cited narratively. Exclusion of non-English full texts may introduce language bias. Publication bias may further underestimate the extent of psychological outcome underreporting.

## 5 Conclusions

Psychological and psychosocial outcomes following digital replantation and revascularization are profoundly underreported in the peer-reviewed hand surgery literature from 2015 to 2025. Only 19 articles met the inclusion criteria and had psychological outcomes, and no study documented formal psychiatric or behavioral health referral rates. The available evidence suggests substantial psychiatric burden, including high rates of PTSD and depression, associations between pre-injury anxiety and surgical failure, adjustment disorder after failed replantation, limited assessment of sleep disturbance, and body image disruption that impacts patients’ lived experiences in ways not captured by traditional outcome measures.

While the hand surgery community has achieved extraordinary technique in replantation, outcome reporting has not fully captured the patient experience of injury and recovery. Future improvements should match technical sophistication with equivalent attention to the patient's experience. Standardized inclusion of validated psychological instruments in replantation outcome reporting and structured integration of behavioral health into replantation care pathways are needed to better characterize and address the long-term effects of injuries in the evolution in the standard of care.

## Declaration of generative AI and AI-assisted technologies in the manuscript preparation process

During the preparation of this work the authors used CLAUDE in order to synthesize literature synthesis, structural organization, and manuscript editing. After using this tool, the authors reviewed and edited the content as needed and take full responsibility for the content of the published article.

## Funding

This research did not receive any specific grant from funding agencies in the public, commercial, or not-for-profit sectors.

## Declaration of competing interests

The authors declare that they have no known competing financial interests or personal relationships that could have appeared to influence the work reported in this paper.
